# *Stolephorus falco* sp. nov. (Teleostei, Clupeiformes, Engraulidae), a new anchovy from Sumatra, Indonesia

**DOI:** 10.3897/zookeys.1270.175032

**Published:** 2026-02-25

**Authors:** Harutaka Hata, Lauren Sallan, Hiroyuki Motomura

**Affiliations:** 1 Macroevolution Unit, Okinawa Institute of Science and Technology, 1919-1 Tancha, Onna-son, Kunigami-gun, Okinawa 904-0495, Japan Macroevolution Unit, Okinawa Institute of Science and Technology Onna-son Japan; 2 The Kagoshima University Museum, 1-21-30 Korimoto, Kagoshima 890-0065, Japan The Kagoshima University Museum Kagoshima Japan

**Keywords:** Actinopterygii, ichthyofauna, *Stolephorus baganensis*, *Stolephorus dubiosus*, taxonomy

## Abstract

A new anchovy species, *Stolephorus falco***sp. nov**., is described based on three museum specimens collected from northern Sumatra, Indonesia. Although the new species resembles *S. baganensis* Delsman, 1931, *S. dubiosus* Wongratana, 1983, *S. taurus* Hata, Lavoué & Motomura, 2022, and *S. tri* (Bleeker, 1852) in having a predorsal scute and a spine on the pelvic scute, it differs from all four of these taxa in having a distinctively deeper body, fewer scales on the longitudinal series, and additional differences in a number of other characters.

## Introduction

The engraulid genus *Stolephorus* Lacepède, 1803 currently comprises 47 valid species (Wongratana 1983, 1987; Whitehead et al. 1988; Wongratana et al. 1999; Kimura et al. 2009b; Hata and Motomura 2018a, 2018b, 2018c, 2018d, 2018e, 2021a, 2021b, 2021c, 2022a, 2022b, 2023a, 2023b, 2024a, 2024b, 2024c; Hata et al. 2019, 2020a, 2020b, 2021, 2022a, 2022b, 2023, 2024a, 2025a, 2025b, 2026; Gangan et al. 2020). Among of them, “spined *Stolephorus*”—species with the predorsal scute and backward spine on pelvic scute (Hata et al. 2022b: figs 2, 3); *S. tri* (Bleeker, 1852), *S. baganensis* Delsman, 1931, *S. dubiosus* Wongratana, 1983, and *S. taurus* Hata, Lavoué & Motomura, 2022—were shown to genetically form a monophyletic group (Hata et al. 2022b: fig. 11; Hata et al. 2024a: fig. 1).

During a subsequent survey of existing *Stolephorus* material, three specimens deposited in the collections of the Zoologisches Museum Hamburg (ZMH) and collected from Sumatra, Indonesia, were identified as belonging to a new taxon of “spined *Stolephorus*”. This new species is characterized by having the deepest body and fewest scales in longitudinal series of the genus, and it features a unique combination of other characters that further distinguish it from both its closest relatives and other *Stolephorus*. The new species is described herein, increasing the number of valid species in the genus to 48.

## Methods

Methods for counts and proportional measurements, shown in Tables 1, 2 and follow Hata and Motomura (2017). All measurements were made with digital calipers to the nearest 0.01 mm. “Pelvic scute” refers to a scute associated with the pelvic girdle, and “prepelvic scute”, “postpelvic scute”, and “predorsal scute” refer to hard, spine-like scutes anterior to the pelvic scute, posterior to the pelvic scute, and anterior to the dorsal-fin origin, respectively. Abbreviations are as follows—**SL**, standard length; **UGR**, **LGR**, and **TGR**, upper-limb, lower-limb, and total gill rakers, respectively, with associated numbers indicating the specific gill arch; **D–P1**, distance from dorsal-fin origin to pectoral-fin insertion; **D–P2**, distance from dorsal-fin origin to pelvic-fin insertion; **D–A**, distance between dorsal- and anal-fin origins; **P1–P2**, distance between pectoral- and pelvic-fin insertions; **P2–A**, distance from pelvic-fin insertion to anal-fin origin. Institutional codes follow Fricke and Eschmeyer (2025). The type specimens of the new species are deposited in **ZMH** (Universität Hamburg, Biozentrum Grindel und Zoologisches Museum, Ichthyology, Hamburg, Germany) and **KAUM** (Kagoshima University Museum, Kagoshima, Japan)

**Table 1.** Meristics of specimens of *Stolephorus falco* sp. nov.

	Holotype	Paratypes	
	ZMH 28689	ZMH 28749	KAUM–I. 220780
Standard length (mm)	58.8	63.3	58.4
Unbranched dorsal-fin rays	3	3	3
Branched dorsal-fin rays	11	11	11
Total dorsal-fin rays	14	14	14
Unbranched anal-fin rays	3	3	3
Branched anal-fin rays	18	18	17
Total anal-fin rays	21	21	20
Unbranched pectoral-fin rays	1	1	1
Branched pectoral-fin rays	10	12	12
Total pectoral-fin rays	11	13	13
Unbranched pelvic-fin rays	1	1	1
Branched pelvic-fin rays	6	6	6
Total pelvic-fin rays	7	7	7
Gill rakers on 1^st^ gill arch (upper)	18	17	18
Gill rakers on 1^st^ gill arch (lower)	23	22	22
Gill rakers on 1^st^ gill arch (total)	41	39	40
Gill rakers on 2^nd^ gill arch (upper)	13	13	14
Gill rakers on 2^nd^ gill arch (lower)	22	20	22
Gill rakers on 2^nd^ gill arch (total)	35	33	36
Gill rakers on 3^rd^ gill arch (upper)	10	10	10
Gill rakers on 3^rd^ gill arch (lower)	12	13	12
Gill rakers on 3^rd^ gill arch (total)	22	23	22
Gill rakers on 4^th^ gill arch (upper)	8	7	7
Gill rakers on 4^th^ gill arch (lower)	10	10	10
Gill rakers on 4^th^ gill arch (total)	18	17	17
Gill rakers on posterior face of 3^rd^ gill arch	4	4	4
Prepelvic scutes	5	5	5
Scale rows in longitudinal series	32	31	32
Transverse scales	8	8	8
Pseudobranchial filaments	16	Damaged	17
Pectoral-fin rays with melanophores	0	0	0

**Table 2.** Morphometrics of specimens of *Stolephorus falco* sp. nov.

	Holotype	Paratypes	
	ZMH 28689	ZMH 28749	KAUM–I. 220780
Standard length (mm; SL)	58.8	63.3	58.4
As % of SL			
Head length	26.9	26.3	27.5
Body depth	28.8	28.9	28.9
Pre-dorsal fin length	56.0	56.2	55.8
Snout tip to pectoral-fin insertion	28.3	28.3	28.5
Snout tip to pelvic-fin insertion	46.4	45.6	45.3
Snout tip to anal-fin origin	66.4	63.5	63.7
Dorsal-fin base length	13.5	13.7	14.3
Anal-fin base length	20.7	22.2	23.5
Caudal-peduncle length	18.2	17.7	16.9
Caudal-peduncle depth	12.6	12.2	13.7
D–P1	38.8	37.7	36.6
D–P2	30.7	29.6	30.5
D–A	29.7	29.3	29.9
P1–P2	20.4	18.6	18.1
P2–A	20.9	19.2	18.7
Pectoral-fin length	18.8	17.7	19.3
Pelvic-fin length	12.0	11.9	12.2
Orbit diameter	8.4	8.2	7.9
Eye diameter	7.3	7.3	7.2
Snout length	3.5	3.2	3.1
Interorbital length	6.5	6.2	6.5
Maxilla length	23.5	damaged	22.8
Mandibular length	18.2	18.2	18.0
Supramaxilla end to maxilla end	6.9	damaged	5.8
Postorbital length	15.8	15.2	16.2

Abbreviations: D–P1: distance from dorsal-fin origin to pectoral-fin insertion; D–P2: distance from dorsal-fin origin to pelvic-fin insertion; D–A: distance between origins of dorsal and anal fins; P1–P2: distance between insertions of pectoral and pelvic fins; P2–A: distance from pelvic-fin insertion to anal-fin origin.

## Taxonomy

### Stolephorus falco sp. nov.

Taxon classificationAnimaliaClupeiformesEngraulidae

066E0BA3-DCFB-5F8A-AC8E-24A5F1B3593F

https://zoobank.org/C807B54C-43F6-4CB1-A847-85FD5EC0F39C

**Figure 1.**
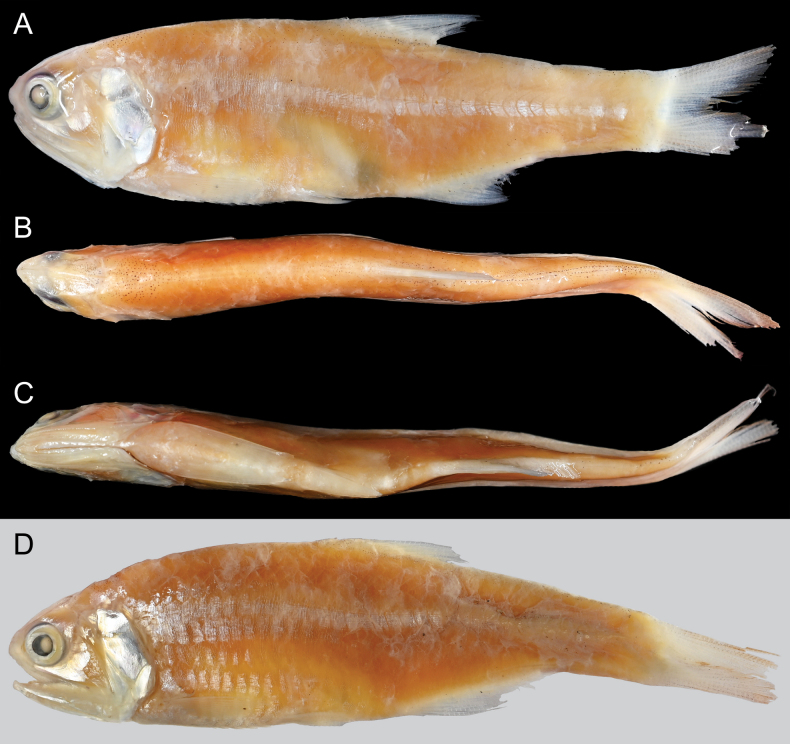
*Stolephorus falco* sp. nov., from Kwalu, Sumatra, Indonesia. **A–C**. Holotype, ZMH 28689, 58.8 mm SL; **A**. Lateral view; **B**. Dorsal view; **C**. Ventral view); **D**. Lateral view of paratype ZMH 28749, 63.3 mm SL.

**Figure 2.**
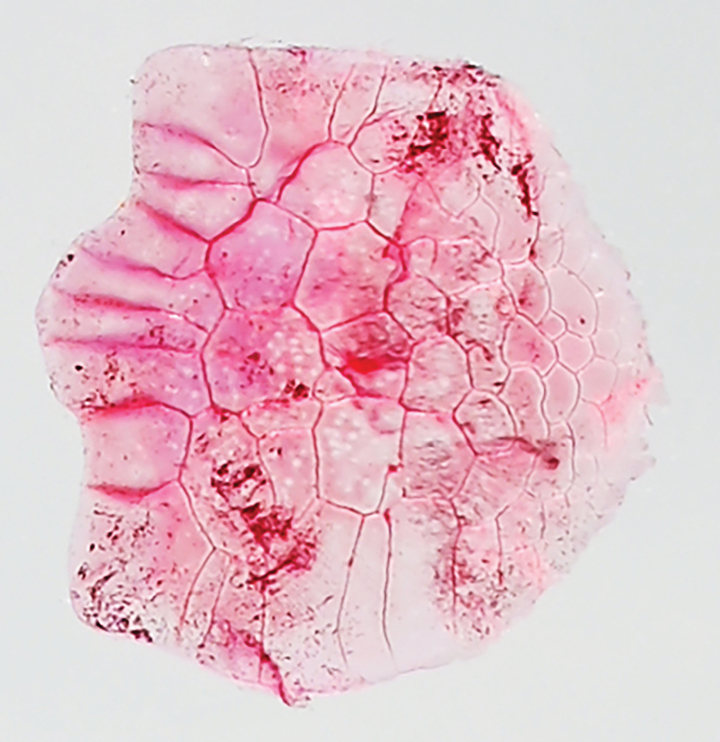
Stained scale removed from right side of midbody (below dorsal fin) of paratype of *Stolephorus falco* sp. nov., ZMH 28749, 63.3 mm SL, from Kwalu, Sumatra, Indonesia (left–right inverted).

Figs 1, 2; Tables 1–4 New English name: Falcon Anchovy

#### Holotype.

• ZMH 28689, 58.8 mm SL, off Kualu, Sumatera Utara, Sumatra, Indonesia, 15 Aug. 1898.

#### Paratypes.

• 2 specimens, 58.4–63.3 mm SL, all specimens collected with the holotype. ZMH 28749, 63.3 mm SL, KAUM–I. 220780 (formerly ZMH 28689), 58.4 mm SL.

#### Diagnosis.

A species of *Stolephorus* with the following combination of characters: 1UGR 17 or 18, 1LGR 22 or 23, 1TGR 39–41; 2UGR 13 or 14, 2LGR 20–22, 2TGR 33–36; 3UGR 10, 3LGR 12or 13, 3TGR 22 or 23; 4UGR 7 or 8 (8), 4LGR 10, 4TGR 17 or 18; scales rows in longitudinal series 31 or 32; maxilla long, 22.8–23.5% of SL, its posterior tip slightly not reaching to posterior margin of opercle; pelvic fin long, 11.9–12.2% of SL, its posterior tip reaching to vertical through 3^rd^ to 4^th^ dorsal-fin ray origin when depressed; paired dark longitudinal lines on dorsum posterior to dorsal fin, but not on anterior to dorsal fin; body scales non-deciduous, with densely reticulated grooves; head large, 26.3–27.5% of SL; body deep, 28.8–28.9% of SL.

#### Description.

Counts and measurements (expressed as percentages of SL) are shown in Tables 1, 2. Data for the holotype are given in parentheses (if different). Body strongly compressed, oblong, deepest at dorsal-fin origin. Dorsal contour elevated from snout tip to dorsal-fin origin, thereafter gently decreasing to uppermost point of caudal-fin base. Ventral profile of body lowering from lower-jaw tip to pelvic-fin insertion thereafter gently rising to lowermost point of caudal-fin base. Abdomen from region between pectoral fins to pelvic-fin insertion covered with 4 spine-like scutes backwardly projecting. Single spine projecting backwardly on pelvic scute. No scutes abdomen before pectoral fins and posterior to pelvic fin. Single spine-like predorsal scutes located just before dorsal-fin origin. Pectoral fin triangular, dorsal, ventral, and posterior margins of fin nearly linear; fin insertion slightly posterior to posterior margin of opercle, lower than level of snout tip; posterior tip of fin pointed, not reaching to pelvic-fin insertion; uppermost ray unbranched, all other rays branched. Elongated triangular axillary scale shorter than pectoral fin; located above pectoral-fin insertion. Pelvic fin triangular, anterior and posterior margins nearly linear; fin insertion anterior to dorsal-fin origin; posterior tip pointed; depressed fin posteriorly reaching to vertical through origin of 3^rd^ (4^th^) dorsal-fin ray; anteriormost fin ray unbranched, all other rays branched. Axillary scale of pelvic fin detached in holotype (elongated triangular axillary scale shorter than pectoral fin; located above pelvic-fin insertion in paratype). Dorsal fin triangular, anterior and posterior profiles nearly linear; fin origin posterior to posteriormost point of pelvic-fin base; three anteriormost rays unbranched, all other rays branched; depressed fin not posteriorly beyond vertical through posteriormost point of anal-fin base. Origin of anal fin located just below 9^th^ (11^th^ or 12^th^) dorsal-fin ray origin; three anterior fin rays unbranched, all other rays branched. Elongated sheath scales on bases of dorsal and anal fins. Caudal fin forked, posterior tips of both lobes pointed, outer contour of each lobe nearly straight (lower part of lower lobe broken in holotype). Anus just in front of anal-fin origin. Snout rounded, projecting beyond lower-jaw tip. Orbit oval, entirely covered with thin adipose eyelid. Eye and pupil round. Nostrils paired close to each other, positioned just anterior to orbit. Mouth large, inferior, oblique to body axis, extending backward to posterior margin of orbit. Maxilla long, posterior tip slightly not reaching to posterior margin of opercle. Lower jaw slender. Single rows of conical teeth on both jaws and palatine. Several conical teeth on vomer. Several rows of conical teeth on pterygoids and dorsal surfaces of basihyal and basibranchial. Single row of fine teeth on dorsal surface of hyoid arch. No tongue. Posterior margin of preopercle round, not intended. No serration on posterior margins of preopercle and opercle. Gill membrane without serrations. Pseudobranchial filaments covered with thin fleshy membrane; length of longest filament less than eye diameter. Gill rakers presenting on inner surfaces of 1^st^ to 4^th^ gill arches and posterior surface of 3^rd^ epibranchial; gill rakers long, slender, and rough. Isthmus muscle long, reaching anteriorly to posterior margin of gill membranes. Urohyal hidden by isthmus muscle. Body scales cycloid, not deciduous, almost remain on type specimens; dense reticulation of grooves on body scales (Fig. 2). No scales on head and fins except for broad, triangular sheath on caudal fin. Lateral line absent.

#### Coloration of preserved specimens.

Body uniformly, light brown. Silver longitudinal band slightly narrower than eye diameter running from upper part of cleithrum to caudal-fin base. Melanophores scattered along dorsal-fin rays and fin bases of dorsal and anal fins. No melanophores on pectora, pelvic, and anal fins. Melanophores scattered on caudal fin-rays, dense on outer margin of fin. Melanophores scattered on posterior margins of scales on upper part of lateral surface of body. Pair of dark patches on parietal region. Melanophores densely gathered on occipital region forming single patch. Paired longitudinal dark lines on dorsum posterior to dorsal fin, but no lines on dorsum anterior to dorsal fin. Lateral surface of head including opercle uniformly silver. Snout and both jaws translucent. Melanophores not scattered on mouth roof, gill rakers, gill arches, gill filaments, and inner surfaces of opercle and hyoid arch (few melanophores scattered on innersurface on hyoid arch in KAUM–I. 220780). Fresh coloration unknown.

#### Distribution.

*Stolephorus falco* sp. nov. is currently known only from Kualu, North Sumatra, Indonesia.

#### Etymology.

The specific name falco is derived from Latin meaning “falcon”, in reference to the talon-like hard spine on the dorsum of the species.

#### Comparisons.

The new species is assignable to the genus *Stolephorus*, as determined by Whitehead et al. (1988) and Wongratana et al. (1999), having a long isthmus muscle anteriorly reaching to the posterior margin of the gill membrane, the urohyal embedded in the isthmus muscle, prepelvic scutes, and the anal-fin origin just positioned below the middle of the dorsal-fin base and lacking of the postpelvic scutes.

**Figure 3.**
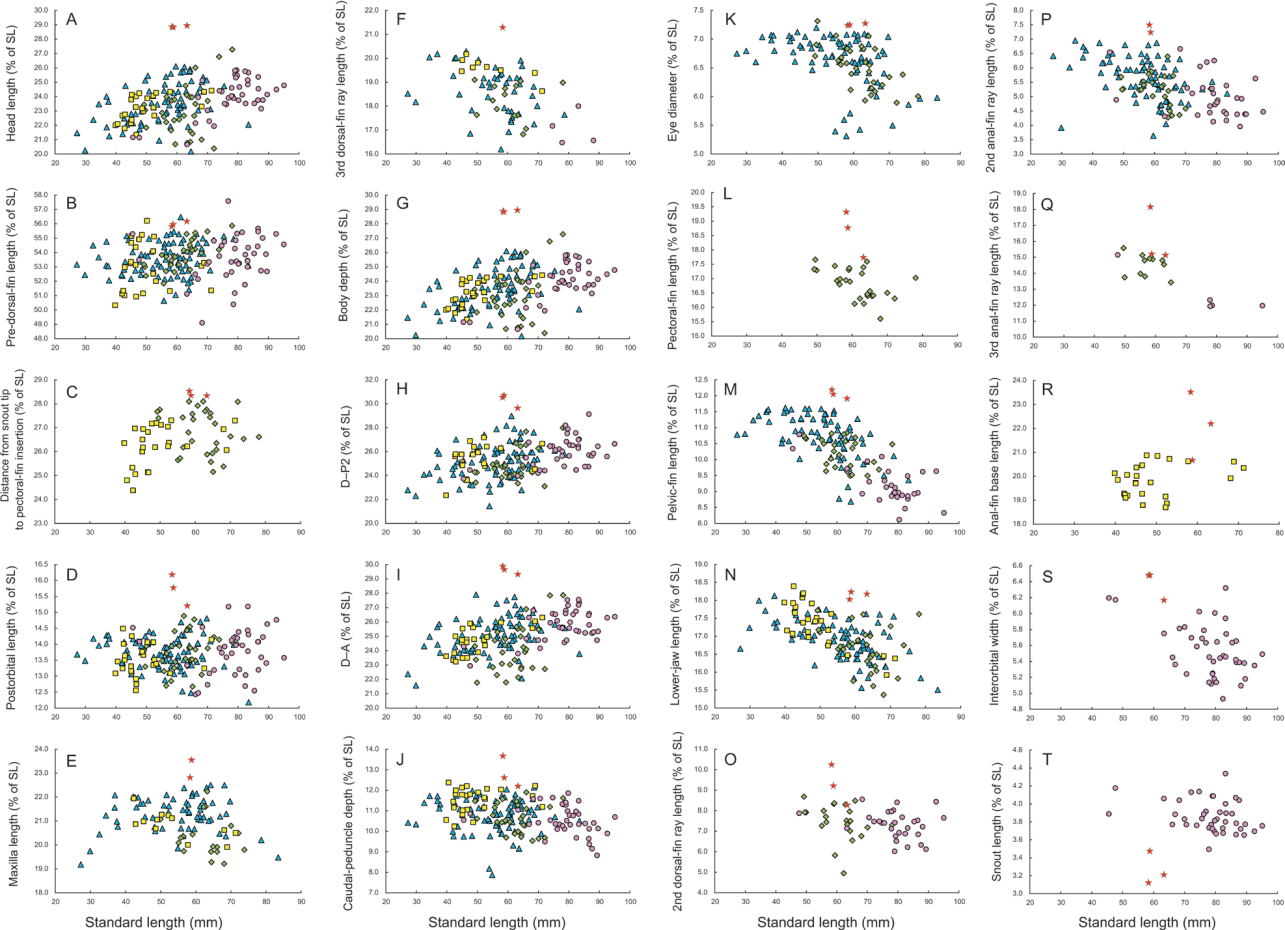
Measurements relative to standard length in *Stolephorus falco* sp. nov. (red stars), *S. baganensis* (blue triangles), *S. dubiosus* (green squares), *S. taurus* (yellow triangles), and *S. tri* (pink circles). All as % of standard length (SL). **A**. Head length; **B**. Pre-dorsal-fin length; **C**. Distance from snout tip to pectoral-fin insertion; Postorbital length; **E**. Maxilla length; **F**. Third dorsal-fin ray length; **G**. Body depth; **H**. Distance from dorsal-fin origin to pelvic-fin insertion (D–P2); **I**. Distance between origins of dorsal and anal fins (D–A); **J**. Caudal-peduncle depth; **K**. Eye diameter; **L**. Pectoral-fin length; **M**. Pelvic-fin length; **N**. Lower-jaw length; **O**. Second dorsal-fin ray length; **P**. Second anal-fin ray length; **Q**. Third anal-fin ray length; **R**. Anal-fin base length; **S**. Interorbital width; **T**. Snout length.

Based on its predorsal scute and backward spine on pelvic scute, *Stolephorus falco* sp. nov. is considered as probable member of the “spined *Stolephorus*” clade (see the Introduction). Among the “spined *Stolephorus*”, the new species is easily distinguished from *S. dubiosus* and *S. taurus* by its fewer gill rakers [1TGR 39–41, 2TGR 33–36, 3TGR 22–23, 4TGR 17–18, and gill rakers on posterior surface of 3^rd^ epibranchial 4 in *S. falco* sp. nov. vs 45–53, 39–48, 26–31, 20–25, and 5–7 in *S. dubiosus*; 45–49, 39–42, 24–28, 19–22, and 5 or 6 (rarely 4 or 7) in *S. taurus*; Table 3], fewer scale rows in longitudinal series (31–32 vs 34–37 in *S. dubiosus*; 33–35 in *S. taurus*; Table 4), longer head (26.3–27.5% of SL vs 23.2–26.4% in *S. dubiosus*; 23.5–26.5% in *S. taurus*; Fig. 3A), pre-dorsal-fin length [55.8–56.2% of SL vs 51.8–55.9% in *S. dubiosus*; 50.3–55.2% (rarely 56.2%) in *S. taurus*; Fig. 3B], distance between snout tip and pectoral-fin insertion (28.3–28.5% of SL vs 25.2–28.1% in *S. dubiosus* vs 24.4–27.3% in *S. taurus*; Fig. 3C), postorbital region (15.2–16.2% vs 12.7–14.9% in *S. dubiosus*; 12.6–14.5% in *S. taurus*; Fig. 3D), maxilla (22.8–23.5% of SL vs 19.2–22.2% in *S. dubiosus*; 19.9–21.9% in *S. taurus*; Fig. 3E), 3^rd^ dorsal-fin ray (21.3% of SL vs 16.8–19.1% in *S. dubiosus*; 18.6–20.2% in *S. taurus*; Fig. 3F) and deeper body (28.8–28.9% of SL vs 20.4–27.3% in *S. dubiosus*; 21.3–24.4% in *S. taurus*; Fig. 3G), D–P2 (29.6–30.7% of SL vs 23.1–28.0% in *S. dubiosus*; 22.3–27.2% in *S. taurus*; Fig. 3H), D–A (29.3–29.9% vs 21.8–27.9% in *S. dubiosus*; 23.2–26.3% in *S. taurus*; Fig. 3I), and caudal peduncle (12.2–13.7% of SL vs 9.5–11.9% in *S. dubiosus* vs 10.2–12.4% in *S. taurus*; Fig. 3J). Moreover, the new species further differs from *S. dubiosus* in having a wider eye diameter (7.2–7.3% of SL vs 5.9–7.3% in *S. dubiosus*; Fig. 3K), pectoral fin (17.7–19.3% of SL vs 15.6–17.7%; 3L), pelvic fin (11.9–12.2% of SL vs 9.5–10.8%; Fig. 3M), lower jaw (18.0–18.2% of SL vs 15.4–17.7%; Fig. 3N), 2^nd^ dorsal-fin ray (8.3–10.2% of SL vs 4.9–8.7%; Fig. 3O), 2^nd^ anal-fin ray (7.2–7.5% of SL vs 4.3–6.4%; Fig. 3P), and 3^rd^ anal-fin ray (15.1–18.2% vs 13.4–15.6%; Fig. 3Q), the pelvic fin posteriorly reaching to vertical through 3^rd^ to 4^th^ dorsal-fin ray origin [vs short of the dorsal-fin origin (rarely reaching to vertical through 1^st^ or 2^nd^ dorsal-fin ray origin) in *S. dubiosus*]. Moreover, anal-fin base length of *S. falco* sp. nov. is longer than that of *S. taurus* (20.7–23.5% of SL in *S. falco* sp. nov. vs 18.7–20.9% in *S. taurus*; Fig. 3R).

**Table 3.** Frequency distribution of total gill rakers on 1^st^, 2^nd^, 3^rd^, 4^th^ gill arch and posterior surface of 3^rd^ epibranchial in *Stolephorus falco* sp. nov., *S. tri*, *S. baganensis*, *S. taurus*, and *S. dubiosus*, based on specimens examined in this study.

Total gill rakers on 1^st^ gill arch																							
		32	33	34	35	36	37	38	39	40	41	42	43	44	45	46	47	48	49	50	51	52	53
*S. falco* sp. nov.	*n* = 3								1	1	1												
*S. tri*	*n* = 41	3	4	8	7	9	4	4	2														
*S. baganensis*	*n* = 81				1	2	6	8	20	18	10	10	5										
*S. taurus*	*n* = 25														4	5	6	5	5				
*S. dubiosus*	*n* = 28														1	2	3	4	7	5	4		2
Total gill rakers on 2^nd^ gill arch																							
		27	28	29	30	31	32	33	34	35	36	37	38	39	40	41	42	43	44	45	46	47	48
*S. falco* sp. nov.	*n* = 3							1		1	1												
*S. tri*	*n* = 41	7	8	14	5	3	4																
*S. baganensis*	*n* = 81					5	7	16	13	21	12	4	3										
*S. taurus*	*n* = 26													7	11	5	3						
*S. dubiosus*	*n* = 28													3	1	3	5	8	2	3	2		1
Total gill rakers on 3^rd^ gill arch																							
		18	19	20	21	22	23	24	25	26	27	28	29	30	31								
*S. falco* sp. nov.	*n* = 3					2	1																
*S. tri*	*n* = 41	5	7	10	5	4																	
*S. baganensis*	*n* = 81			2	9	31	17	21	2														
*S. taurus*	*n* = 26							1	4	13	7	1											
*S. dubiosus*	*n* = 28									1	6	12	4	5	1								
Total gill rakers on 4^th^ gill arch																							
		15	16	17	18	19	20	21	22	23	24	25											
*S. falco* sp. nov.	*n* = 3			2	1																		
*S. tri*	*n* = 41	2	20	13	5	1																	
*S. baganensis*	*n* = 82		5	16	39	17	4	1															
*S. taurus*	*n* = 26					2	16	7	1														
*S. dubiosus*	*n* = 29						2	8	11	3	4	1											
Gill rakers on posterior surface of 3^rd^ epibranchial																							
		3	4	5	6	7																	
*S. falco* sp. nov.	*n* = 3		3																				
*S. tri*	*n* = 41		7	32	2																		
*S. baganensis*	*n* = 82	2	30	41	9																		
*S. taurus*	*n* = 26		4	14	7	1																	
*S. dubiosus*	*n* = 29			13	13	3																	

**Table 4.** Frequency distribution of scale rows in longitudinal series in *Stolephorus falco* sp. nov., *S. tri*, *S. baganensis*, *S. taurus*, and *S. dubiosus*, based on specimens examined in this study.

		Scale rows in longitudinal series						
		31	32	33	34	35	36	37
*S. falco* sp. nov.	*n* = 3	1	2					
*S. tri*	*n* = 40			7	17	9	7	
*S. baganensis*	*n* = 81			2	37	29	12	1
*S. taurus*	*n* = 25			6	17	2		
*S. dubiosus*	*n* = 30				8	14	7	1

Additionally, the new species differs from *S. tri* in having more gill rakers [1TGR 39–41 and 2TGR 33–36 in *S. falco* sp. nov. vs 32–38 (rarely39) and 27–32 in *S. tri*; Table 3), fewer scale rows in longitudinal series (31–32 vs 33–36; Table 4), longer pre-dorsal-fin length [55.8–56.2% of SL vs 49.1–55.5% (rarely 57.6%); Fig. 3B], pelvic fin (11.9–12.2% of SL vs 8.1–10.8%; Fig. 3M), postorbital region (15.2–16.2% of SL vs 12.4–15.2%; Fig. 3D), interorbital region (6.2–6.5% of SL vs 4.9–6.3%; Fig. 3S), 2^nd^ dorsal-fin ray (8.3–10.2% of SL vs 6.0–8.6%; Fig. 3O), 3^rd^ dorsal-fin ray (21.3% of SL vs 16.5–18.0%; Fig. 3F), 2^nd^ anal-fin ray (7.2–7.5% of SL vs 4.0–6.7%; Fig. 3P), and 3^rd^ anal-fin ray (15.1–18.2% vs 11.9–15.2%; Fig. 3Q), shorter snout (3.1–3.5% of SL vs 3.5–4.3%; Fig. 3T), deeper body (28.8–28.9% of SL vs 20.7–25.8%; Fig. 3G), D–P2 (29.6–30.7% of SL vs 23.7–29.1%; Fig. 3H), D–A (29.3–29.9% vs 22.9–27.6%; Fig. 3I), and caudal peduncle (12.2–13.7% of SL vs 8.8–11.9%; Fig. 3J), the pelvic fin posteriorly reaching to vertical through 3^rd^ to 4^th^ dorsal-fin ray origin (vs short of the dorsal-fin origin). Dorsal pigmentation from the occipital area to the dorsal-fin origin also separates *S. falco* sp. nov. (no dark lines; Fig. 1B) and *S. tri* (usually paired dark lines; Hata et al. 2019: fig. 19).

*Stolephorus falco* sp. nov. most closely resembles *S. baganensis*, as the former was previously identified as the latter in by Hata et al.’s (2022b: 12) identification keys on the basis of published information. However, on direct examination of the type specimens, the new species is clearly separated from *S. baganensis* by longer head [26.3–27.5% of SL vs 22.3–26.7% (< 26% in specimens > 50 mm SL) in *S. baganensis*; Fig. 3A], pre-dorsal-fin length [55.8–56.2% of SL vs 50.6–55.4% (rarely 56.5%, only 1 of 82 specimens); Fig. 3B], eye diameter (7.2–7.3% of SL vs 5.3–7.2%; Fig. 3K), pelvic fin (11.9–12.2% of SL vs 8.7–11.6%; Fig. 3M), postorbital region (15.2–16.2% vs 12.2–14.8%; Fig. 3D), maxilla (22.8–23.5% of SL vs 19.2–22.5%; Fig. 3E), lower jaw (18.0–18.2% of SL vs 15.5–18.1%; Fig. 3N), and 3^rd^ dorsal-fin ray (21.3% of SL vs 16.2–20.3%; Fig. 3F), 2^nd^ anal-fin ray (7.2–7.5% of SL vs 3.6–7.0%; Fig. 3P), deeper body (28.8–28.9% of SL vs 20.2–26.1%; Fig. 3G), D–P2 (29.6–30.7% of SL vs 21.4–29.0%; Fig. 3H), D–A (29.3–29.9% of SL vs 21.6–27.9%; Fig. 3I), and caudal peduncle depth (12.2–13.7% of SL vs 7.9–12.1%; Fig. 3J), and fewer scale rows in longitudinal series (31 or 32 vs 33–37; Table 4).

In addition, the new species has the deepest body of all species of the genus (the second largest species is *S. dubiosus*, whose body depth reaches 27.3% of its length; Hata et al. 2022b), but it also features among the fewest scale rows in longitudinal series (the second is *Stolephorus brachycephalus* Wongratana, 1983, a species with 32–34 scales; Hata and Motomura 2024c).

#### Remarks.

Because *S. falco* is here named from only three known specimens collected from Kwalu (northeastern Sumatra), its exact distribution is unknown. However, the new species has never been recorded in the ichthyofaunal surveys in neighboring areas such as Thai Andaman Sea (Matsuura and Kimura 2005; Kimura et al. 2009a; Satapoomin 2011; Hata et al. 2024b); Penang on the west coast of the Malay Peninsula (Lavoué et al. 2022), Johor Strait at the southern tip of the Malay Peninsula (Kimura et al. 2015), east coast of Malay Peninsula (Matsunuma et al. 2011; Motomura et al. 2021; Giat et al. 2021), and eastern Indonesia (White et al. 2013). It is, therefore, likely to be endemic to the vicinity of the type locality in Sumatra.

#### Comparative materials.

*Stolephorus baganensis*: 82 specimens, 27.3–83.5 mm SL: listed by Hata et al. (2022b) and 51 additional specimens: MNHN 2001-3142, 83.5 mm SL, off Vung Tau, Vietnam; MZB 28362, 78.6 mm SL, off Tanjung Pasir, Java, Indonesia (obtained at Tanjung Pasir Fish Market), NMMB-P32374, 22 specimens, 41.9–63.6 mm SL, Matang, Perak, Malaysia; QM I. 28329, 50.5 mm SL, Lassa River estuary, Kuala Matu, Sarawak, Borneo, Malaysia; RMNH.PISC.85266, 2 specimens, paralectotypes of *Engraulids tri*, 66.8–75.4 mm SL, Indonesia; USMFC (82) 00078, 62.5 mm SL, Batu Lintang, Merbok estuary, Kedah, Malaysia (5°37'26.4"N, 100°23'39.1"E); USNM 316674, 2 specimens, 62.2–66.9 mm SL, Vung Tau, Ba Ria Vung Tau, Vietnam (obtained at fish market in Vung Tau); ZMUC 41, 48.4 mm SL, Rangoon, Myanmar; ZMH 10675, 4 of 11 specimens, 34.4–43.1 mm SL, Kuala Langat, Selangor, Malaysia; ZMH 10676, 4 of 8 specimens, 37.5–50.9 mm SL, Kuala Selangor, Selangor, Malaysia; ZRC 39790, 12 specimens, 42.7–68.7 mm SL, Matang, Perak, Malaysia. *Stolephorus dubiosus*: 31 specimens, 49.2–78.1 mm SL: listed by Hata et al. (2022b) and 8 additional specimens, ANSP 61760, paratypes of *Stolephorus dubiosus*, 2 specimens, 49.5–58.1 mm SL, estuary of Chao Phraya River, Paknam, Thailand; BMNH 1989.2.2.74–75, 2 specimens, 49.2–59.2 mm SL, off Semarang, Java, Indonesia, 10 m depth; BMNH 1981.7.29.246, 64.2 mm SL, Takisung, Tanah Laut, Kalimantan, Indonesia; RMNH.PISC.85265, 3 specimens, paralectotypes of *Engraulids tri*, 64.9–73.8 mm SL, Indonesia; ZMA 108.373, 56.6 mm SL, Soerabaja, Java, Indonesia. *Stolephorus taurus*: 26 specimens, 39.8–71.3 mm SL: listed by Hata et al. (2022b) and 6 additional specimens, BMNH 1889.2.1.1840, paratype of *Stolephorus dubiosus*, 68.1 mm SL, Orissa, India; BMNH 1969.4.22.1803, 71.3 mm SL, Godavari Estuary, Andhra Pradesh, India; MNHN 992, 1 of 3 specimens, 57.7 mm SL, Ganges, India; RMNH.PISC.8587, 68.9 mm SL, Orissa, India; ZMUC P.18273–18274, 2 specimens, 45.2–47.6 mm SL, Tharangambadi, Tamil Nadu, India. *Stolephorus tri*: 41 specimens, 45.5–95.1 mm SL: listed by Hata et al. (2022b) and 14 additional specimens, BC 63-1348, 77.9 mm SL, Muar Market, Johor, Malaysia; BMNH 1977.11.30.163–166, 2 of 4 specimens, 88.1–92.6 mm SL, Surat Thani Province, Thailand; RMNH.PISC.17735, 89.6 mm SL, Pulau Weh, northern Sumatra, Indonesia; RMNH.PISC.24961, 6 specimens, paralectotypes of *Engraulids tri*, 72.2–86.5 mm SL, Indonesia; UMMZ 225767, 73.9 mm SL, approx. 27 km southeast of estuary of Hau River (Mekong Basin), Vietnam (9°14'42.0"N, 106°17'36.0"E); USMFC (82) 00031, 80.2 mm SL, Sungai Batu, Malaysia; USMFC (82) 00082, 82.3 mm SL, USMFC (82) 00083, 78.0 mm SL, Batu Maung fish landing port, Penang, Malaysia.

## Supplementary Material

XML Treatment for Stolephorus falco

## Additional information

### Use of AI

No use of AI was reported.

### Author ORCIDs

Harutaka Hata https://orcid.org/0000-0002-6996-3628

Lauren Sallan https://orcid.org/0000-0002-4441-1456

Hiroyuki Motomura https://orcid.org/0000-0002-7448-2482

### Data availability

All of the data that support the findings of this study are available in the main text.
